# Growth in marine mammals: a review of growth patterns, composition and energy investment

**DOI:** 10.1093/conphys/coad035

**Published:** 2023-06-21

**Authors:** Stephanie K Adamczak, Elizabeth A McHuron, Fredrik Christiansen, Robin Dunkin, Clive R McMahon, Shawn Noren, Enrico Pirotta, David Rosen, James Sumich, Daniel P Costa

**Affiliations:** Ecology and Evolutionary Biology Department, University of California Santa Cruz, 130 McAlister Way, Santa Cruz, CA 95064, USA; Cooperative Institute for Climate, Ocean, and Ecosystem Studies, University of Washington, 3737 Brooklyn Ave NE, Seattle, WA 98105, USA; Department of Ecoscience – Marine Mammal Research, Aarhus University, Frederiksborgvej 399, 4000 Roskilde, Denmark; Ecology and Evolutionary Biology Department, University of California Santa Cruz, 130 McAlister Way, Santa Cruz, CA 95064, USA; Sydney Institute of Marine Science, 9 Chowder Bay Road, Mosman, NSW 2088, Australia; Institute of Marine Science, University of California Santa Cruz, Santa Cruz CA, USA; Centre for Research into Ecology and Environmental Modelling, University of St. Andrews, St. Andrews, KY16 9LZ, UK; Marine Mammal Research Unit, Institute for the Oceans and Fisheries, University of British Columbia, 2022 Main Mall, Vancouver, BC V6T 1Z4, Canada; Fisheries, Wildlife, and Conservation Science Department, Oregon State University, Hatfield Marine Science Center, 2030 SE Marine Science Driver, Newport, Oregon 97365, USA; Ecology and Evolutionary Biology Department, University of California Santa Cruz, 130 McAlister Way, Santa Cruz, CA 95064, USA; Institute of Marine Science, University of California Santa Cruz, Santa Cruz CA, USA

**Keywords:** marine mammal, growth, body size

## Abstract

Growth of structural mass and energy reserves influences individual survival, reproductive success, population and species life history. Metrics of structural growth and energy storage of individuals are often used to assess population health and reproductive potential, which can inform conservation. However, the energetic costs of tissue deposition for structural growth and energy stores and their prioritization within bioenergetic budgets are poorly documented. This is particularly true across marine mammal species as resources are accumulated at sea, limiting the ability to measure energy allocation and prioritization. We reviewed the literature on marine mammal growth to summarize growth patterns, explore their tissue compositions, assess the energetic costs of depositing these tissues and explore the tradeoffs associated with growth. Generally, marine mammals exhibit logarithmic growth. This means that the energetic costs related to growth and tissue deposition are high for early postnatal animals, but small compared to the total energy budget as animals get older. Growth patterns can also change in response to resource availability, habitat and other energy demands, such that they can serve as an indicator of individual and population health. Composition of tissues remained consistent with respect to protein and water content across species; however, there was a high degree of variability in the lipid content of both muscle (0.1–74.3%) and blubber (0.4–97.9%) due to the use of lipids as energy storage. We found that relatively few well-studied species dominate the literature, leaving data gaps for entire taxa, such as beaked whales. The purpose of this review was to identify such gaps, to inform future research priorities and to improve our understanding of how marine mammals grow and the associated energetic costs.

## Teaser

Marine mammal body size, body composition and growth rates of individuals are important proxies for individual and population health. There remain gaps in our understanding of growth trajectories, tissue composition and energy allocation to growth in marine mammals. We conducted a literature review to address these knowledge gaps and identify future research priorities.

## Introduction

Body size is an important driver of individual survival and reproductive success, and drives population and species life history (Blueweiss *et al.,* 1978; Lindstedt and Calder, 1981; Lindstedt and Boyce, 1985; Calder, 2001). Being structurally large can provide direct benefits, such as increased performance in intra-specific competition and defense from predation, and energetic benefits, such as decreased mass-specific metabolic costs and increased energy storage (Bartholomew, 1970; Kleiber, 1975; McNab, 1980; Lindstedt and Boyce, 1985; Millar and Hickling, 1990; Williams, 1999; Molnár *et al.,* 2009; Gunnlaugsson *et al.,* 2020). The capacity of large individuals to deposit and utilize body energy reserves can greatly influence a species’ ability to survive periods of low food availability, and to exploit spatially and temporally variable resources (Lindstedt and Boyce, 1985; Costa and Maresh, 2022). Large body size confers a benefit in an aquatic environment by buffering against costs associated with thermal conductivity of water and buffering against extended periods without food due to the ephemeral nature of prey resources. As such, marine mammals often allocate large amounts of resources to growth of structural size early in life, despite the increased energetic cost of growth, and continue to allocate energy to reserves through adulthood (Christiansen *et al.,* 2022a).

Given the benefits of large body size, neonatal and young animals are at a disadvantage until mature body size is attained. Thus, rapid changes in body size would be expected early in life. Indeed, mammalian growth occurs in two phases: (1) the early life phase when structural lean tissue is primarily deposited, and (2) the physical maturity phase when there is a transition to the deposition of energy stores primarily in the form of lipids (Guenther *et al.,* 1965; Crocker *et al.,* 1998). The initial growth phase determines the asymptotic size of an animal, both in length and mass (McLaren, 1993). In contrast, the second growth phase is characterized by fluctuations in mass and overall body condition, often related to seasonal resource availability of the species’ reproductive cycle, with very minimal fluctuations in structural size (McLaren, 1993; Rosen *et al.,* 2021). Energy allocation to both the primary and secondary growth phases varies in response to intrinsic and extrinsic factors, such as energy requirements and prey availability, that influence individual growth rates, size at physical maturity and body condition. Alterations to growth investment can have lasting effects on an animal’s biology and physiology and lead to population-level impacts due to the repercussions of body size on survival and reproduction (Craig and Ragen, 1999; Pomeroy *et al.,* 1999; Crocker *et al.,* 2001; McMahon *et al.,* 2017).

The application of body size and condition metrics to marine mammal and ecosystem conservation and management requires knowledge of how energy is allocated to deposition of structural and reserve tissues, the costs associated with growth and the factors that influence growth. Improved knowledge of growth processes and energetics can be particularly beneficial for bioenergetic modeling, which explores how energy is metabolized and allocated to various aspects of maintenance, growth and reproduction. Bioenergetic models have been used to assess how and when anthropogenic disturbances that affect energy budgets result in population-level impacts (Costa, 2012; Pirotta *et al.,* 2018a; Keen *et al.,* 2021; Pirotta, 2022). These population-level impacts are a consequence of decreased foraging opportunities, which initially result in reduced investment into non-essential metabolic processes such as growth and reproduction or, ultimately, mortality due to starvation. Accurate quantifications of the costs associated with growth and the factors influencing growth are necessary to improve forecasting via bioenergetic models.

In this review, we synthesize the available literature on marine mammal growth. We aim to address five major themes regarding growth in marine mammals: (1) how marine mammals grow, (2) composition of growth, (3) energetic costs and allocation priorities, (4) empirical estimate of growth costs and (5) factors influencing total body size and energy reserves. Given how important acquiring, storing and using resources are in determining vital rates and individual health, we identify data gaps and potential areas for future research.

## Methods

We used Google Scholar with the search terms ‘bioenergetics’, ‘tissue composition’, ‘muscle composition’, ‘blubber composition’, ‘muscle lipid content’, ‘muscle protein content’, ‘blubber lipid content’, ‘blubber protein content’, ‘growth curve’, ‘growth trajectory’, ‘growth cost’ and ‘energy allocation to growth’ with a combination of ‘marine mammal’, species scientific names and species common names. Additionally, we searched for ‘organohalogen’, ‘organochlorine’ and ‘toxicology’, with a combination of ‘marine mammal’, species scientific names and species common names as we found this literature to be rich in tissue composition data. While we searched across all marine mammal taxa, our focus was on cetaceans and pinnipeds as these are the most studied and speciose marine mammal taxa.

In addition to the literature review, we used existing data to address two issues that have not been well investigated in the literature: (1) the influence of species and life history stages on muscle protein content in cetaceans and (2) the cost of growth in marine mammals. We used Kruskal-Wallis tests to examine differences in muscle protein content reports between cetacean taxonomic groups (*n* = 49 and 5 for mysticetes and odontocetes, respectively), age classes (for mysticetes only, *n* = 7 and 5 for immature and mature, respectively, excluding reports that clumped data across multiple age classes and reproductive statuses) and sex both across age classes and for mature individuals only (for mysticetes only: 24 and 6 for males and females of all age classes, respectively; and 2 each for mature males and females; McKnight and Najab, 2009).

To estimate the cost of growth, defined as the energy required to synthesize and deposit tissues, in marine mammals, we assessed the relationship between mass deposition rate and resting metabolic rate (measured during regular health examinations) for female juvenile northern fur seals (*Callorhinus ursinus*; *n* = 6), adult male bottlenose dolphins (Tursiops truncatus; *n* = 2) and a single male gray whale calf (*Eschrichtius robustus*), all managed in human care. Our methods mirrored those used to estimate the cost of growth in domestic cattle and lab rats (Rattray and Joyce, 1976). For northern fur seals and bottlenose dolphins, we used respirometry and mass measurements from health assessments conducted at uneven intervals. The gray whale data used food intake as a proxy for metabolic needs. Mass deposition rate was determined as the mass change (g) between examination dates divided by the number of days between examinations. We used Wilcoxon signed rank tests to compare resting metabolic rate in positive growth phases (when mass was gained) and negative growth phases (when mass was lost) for both bottlenose dolphins and northern fur seals to test if resting metabolic rate increased while depositing tissues. To estimate the cost of growth in marine mammals we used the slope derived from a linear model testing the relationship between mass deposition and metabolic rate (Rattray and Joyce, 1976). A combination of response variables, including resting metabolic rate and mass-specific metabolic rate, and explanatory variables, including mass deposition rate and mass deposition rate normalized by body size, were tested in the model. When more than one test subject was available we tested the impact of individuals as random effects with linear mixed effect models (Oberg and Mahoney, 2007). We used Akaike Information Criterion (AIC) to select the most parsimonious model (Portet, 2020). Separate models were constructed for each species to account for differences in age and methods used to estimate metabolic rate. The results of these analyses can be found in the ‘Empirical estimate of growth costs’ section. The remaining sections summarize the results of the literature review.

### How do marine mammals grow?

**Figure 1:**
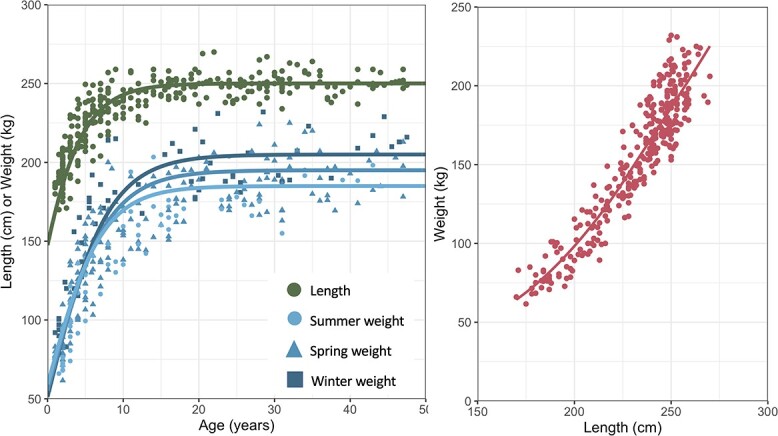
Example of length-at-age, weight-at-age (left panel) and weight-at-length (right panel) relationships derived from data on bottlenose dolphins (*T. truncatus*) from the Sarasota Dolphin Research Program. The weight-at-age data demonstrates seasonal fluctuations in mass only in the secondary growth phase, which is not typically captured in weight-at-age curves; meanwhile during the primary growth phase there seems to be no seasonal fluctuations in mass.

Marine mammals exhibit diminishing structural growth throughout their lifetime, marked by rapid growth prior to physical maturity that slows around adulthood. They are thought to have a finite growing period, causing total body size (or structural size) to reach an asymptote (see below for exceptions). The initial growth phase determines the overall size of an animal, both in length and mass, and includes some fluctuations in mass due to the mobilization and deposition of reserves (Rosen *et al.,* 2021). In contrast, the second growth phase focuses almost entirely on fluctuations in mass due to changes in reserves mediated by season, reproductive status and prey availability (e.g. Lockyer, 1995; Gallagher *et al.,* 2021; Rosen *et al.,* 2021). These growth stages can be described using growth curves that model the length- and weight-at-age, as well as weight-at-length relationships (Figure 1).

Growth curves are typically constructed by fitting a Gompertz, von Bertalanffy or Richards growth model to data on length- or weight-at-age or a regression of weight-at-length (Zeide, 1993; Tjørve and Tjørve, 2017; Hall *et al.,* 2019; Christiansen *et al.,* 2022a). The various stages of growth may not fully be captured by these models, and it may be necessary to construct multiple growth curves for different age classes (e.g. McLaren, 1993; Chabot and Stenson, 2002; Agbayani *et al.,* 2020; Fortune *et al.,* 2021). In particular, growth during the first year of life is often rapid, necessitating a separate model for this life stage (Best and Schell, 1996; Fortune *et al.,* 2021; Rosen *et al.,* 2021). In some cases, males may undergo growth spurts prior to physical maturity that further complicate growth rates for young individuals (e.g. Winship *et al.,* 2001). Additionally, growth models often model asymptotic growth, although research indicates that some species may continue to grow into adulthood, such as mysticetes (Payne, 1979). Such taxonomic groups may not reach an asymptotic size, despite reduced growth rates in adulthood. However, this result may be an artifact of using data from whaled or harvested individuals (see below). Lastly, some species may demonstrate nuanced growth patterns that cannot be represented by a single growth curve. For example, newly weaned bowhead whales (*Balaena mysticetus*) enter a diapause stage where structural growth is halted for three to four years (Schell *et al.,* 1989).

**Figure 2:**
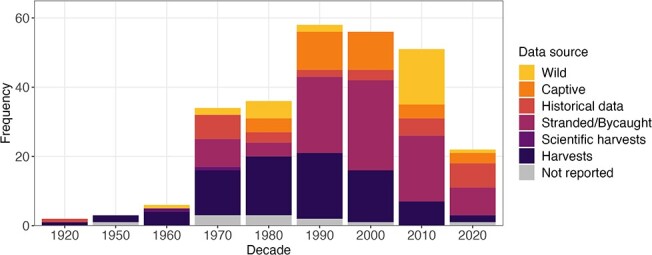
Sources of data used to construct marine mammal growth curves collated in this review with respect to era and data source (*n* = 277). Some growth curves were constructed using two data sources, but the method with the greatest human impact on the population was presented here. For example, if a paper listed their data sources as harvested and stranded animals, we reported this as a harvest. Historical data includes the use of previously published data and museum specimens, and harvests include commercial and subsistence hunts.

There are limitations in the data sources used to construct growth curves, particularly with respect to cetaceans. Most cetacean growth curves have been derived from stranded, bycaught or harvested individuals (Figure 2). Such data may be biased, as stranded individuals may be in poor health and whalers targeted larger individuals, resulting in under- and overestimates, respectively, of size at a given age (e.g. Stevick, 1999). Despite these limitations, these observations do provide valuable bioenergetic information (Irvine *et al.,* 2017). Most growth curves collated here were published 20 or more years ago, with the largest proportion of growth curves published between 1990–2010 (Figure 2). Since then, novel technologies have been developed that facilitate data collection using non-lethal methods, warranting a re-analysis of previously constructed curves. For example, the inter-pulse interval of echolocation clicks has been used to determine sperm whale (*Physeter macrocephalus*) length (Dickson, 2020) and aerial- or laser-photogrammetry has been used to estimate length, mass, and volume of free-living animals (e.g. Christiansen *et al.,* 2018; van Aswegen *et al.,* 2019; Fortune *et al.,* 2021). Although using non-lethal methods may reduce sample size due to limitations of accessing wild animals and the small number of individuals that can be held in human care, there are opportunities to collate data from multiple sources to improve sample size (e.g. Clark *et al.,* 2000).

Availability of growth curves and, therefore, our understanding of marine mammal growth patterns, is highly species-dependent. Data are plentiful for pinnipeds; 100% of phocid seals and 79% of extant otariid species have documented length-at-age, weight-at-age or weight-at-length curves, although, when considering just weight-at-age curves, phocid coverage decreases to just over 50% (Supplementary Table A). Cetacean growth curves are less common due to their fully aquatic lifestyle. For mysticetes, 73% of known species have documented length-at-age, weight-at-age or weight-at-length curves and only 47% of species have weight-at-age curves. Approximately 52% of odontocete species have length-at-age curves, which decreases to 26% when considering just weight-at-age curves (Supplementary Table A). Although some odontocete families have complete coverage of length-at-age, weight-at-age and weight-at-length curves, such as the Kogiids and Monodontiids, there is very poor coverage among the most speciose odontocete family, the Delphinids and little or no data among the beaked whales and river dolphins (Supplementary Table A).

Growth curves documenting lean mass are much sparser than those discussed above. To obtain these measurements the animal must either be killed or isotopic methods must be used, which requires repeated access to the animal. This limits the species for which lean mass growth curves are available to small and/or partially aquatic species. Although lean mass is an important determinant of resting metabolic rate (Rea and Costa, 1992), the use of mass curves to calculate resting metabolic rate likely provides an adequate estimation of metabolic rate for bioenergetic modeling.

### Composition of growth

#### Tissue synthesis

The chemical composition of tissues depends on the form of chemical energy ingested, which allows for the synthesis of various types of tissues. In most vertebrates, the synthesis of new tissues relies on the ingestion of three primary organic macromolecules: proteins, carbohydrates and lipids. Most marine mammals synthesize carbohydrates primarily from protein because they are minimally present in the diet of most species, except for sirenians. Processing ingested lipids into energy reserves is more efficient than processing and depositing protein (Rattray and Joyce, 1976). However, the extent of protein or lipid deposition depends on both the amount and composition of macronutrient intake and growth priorities at that particular life stage.

To synthesize lean muscle tissue, protein intake must exceed protein degradation (Reeds *et al.,* 1982; Fuller and Chen, 1997). Protein intake determines lean mass deposition in marine mammals, although this has predominantly been studied in phocids (Kirsch *et al.,* 2000; Trumble *et al.,* 2003). The deposition of lean muscle tissue has an asymptotic relationship with increasing energy input, as there is a limit to how much lean mass can be deposited within a given timeframe (Fuller and Chen, 1997). In mammals, metabolized protein is excreted as nitrogenous waste in the form of urea (Reeds *et al.,* 1980; Costa *et al.,* 2013), while protein not metabolized can be deposited as structural tissue, and may also be used later as an energy source (Crocker *et al.,* 1998).

When lipids (i.e. triglycerides or wax esters) are ingested, they are broken down into fatty acids and transported through the bloodstream. If energy intake exceeds energy expenditure, these lipids are deposited in adipocytes as energy reserves. Although it was previously thought that wax esters were largely indigestible by mammals, it appears that mysticetes possess the ability to assimilate 99% of dietary wax esters potentially mediated by symbiotic gut microbes (Swaim *et al.,* 2009; Koopman, 2018). In contrast, odontocetes appear to biosynthesize wax esters rather than incorporate dietary wax esters (Koopman, 2018). With respect to triglyceride assimilation, the efficiency of converting ingested lipids to reserve lipids appears to be modulated by diet composition and may be species-specific. In harbor seals (*Phoca vitulina*), lipid and protein digestibility declined on a high lipid diet (Trumble *et al.,* 2003), while in northern fur seals (*C. ursinus*) lipid digestibility improved with moderate to high lipid ingestion (Diaz Gomez *et al.,* 2020). Lipid-poor diets cause a reduction in lipid reserves when an animal is nutritionally challenged (Rosen and Trites, 2005). Thus, efficient tissue deposition and energy storage rely on an appropriate balance in diet composition.

#### Tissue composition

Tissues are primarily comprised of protein, lipid, water and carbohydrates. For most vertebrates, the chemical composition of skeletal muscle is about 70–80% water, 20–30% protein and 1–2% lipid (Anghihan *et al.,* 1969; Kim, 1974; Listrat *et al.,* 2016). The protein content of marine mammal muscle is on par with those reported for other vertebrates, ranging between approximately 18.2–26.9% (fin whale [*Balaenoptera physalus*], sei whale [*Balaenoptera borealis*] and minke whale [*Balaenoptera acutorostrata*]; Lockyer *et al.,* 1985; Víkingsson *et al.,* 2013; bowhead whale [*B. mysticetus*]; O’Hara *et al.,* 2004; sperm whale; Watanabe and Suzuki, 1950; harp seal [*Pagophilus groenlandicus*] and hooded seal [*Cystophora cristata*]; Brunborg *et al.,* 2006; Cape fur seal [*Arctocephalus pusillus*]; Koep *et al.,* 2007). We found no significant trends in muscle protein content between taxonomic groups, age class or sex amongst age classes or for mature individuals (Kruskall-Wallis test; *P* = 0.08; *P* = 0.83; *P* = 0.39; *P* = 0.32, respectively), although our sample was biased towards female mysticetes.

Skeletal muscle lipids include structural lipids and phospholipids that are necessary to build this tissue, in addition to storage lipids that are deposited and mobilized with energetic needs. Because of the additional storage lipids, it is difficult to determine baseline lipid content (i.e. the lipid content consisting of phospholipids and structural lipids required simply to build this tissue) for marine mammals. Estimates of baseline muscle lipid content may be obtained from fasted animals that have depleted their energy reserves, such as capital breeding marine mammals; however, no such data are currently publicly available. Given the lack of data, the minimum reported value of muscle lipid content, 0.1% in the short-beaked common dolphin (*Delphinus delphis*; Lazar *et al.,* 2012), may represent the best available value for baseline muscle lipid content. Baseline muscle lipid content is important for accurately determining the energetic cost of structural growth (see Energy allocation to growth and energetic costs section). However, it should be noted that the above minimum value for muscle lipid content was derived from the ecotoxicology literature and the age, sex or nutritional condition of the study animal was not provided.

**Figure 3:**
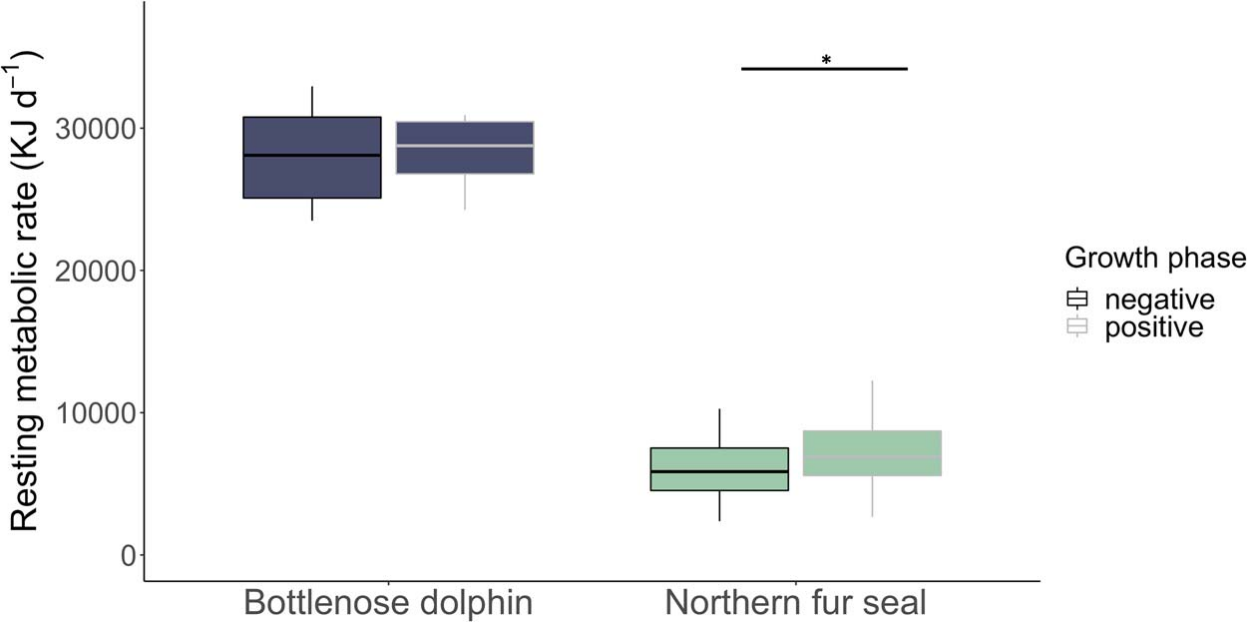
Resting mass-specific metabolic rate (kJ d^−1^) in negative and positive growth phases for juvenile bottlenose dolphins (*n* = 2) and northern fur seals (*n* = 6). Vertical line indicates statistically significant difference from Wilcoxon signed rank test results (*P* = 2.4 × 10^−3^). Data contributed by the University of California Santa Cruz Long Marine Laboratory and the University of British Columbia Marine Mammal Research Unit.

Additional lipids in skeletal muscle are considered energy reserves, and as such typically take the form of triglycerides (Young, 1976; Trumble *et al.,* 2010). In fin whales and Weddell seals (*Leptonychotes weddellii*), muscle lipid content appears to vary in proportion to the lipid content of blubber (the primary energy reserve tissue), suggesting that muscle lipid content reflects energy storage levels (see Figure 1 in Lockyer, 1986; Figure 1 in Trumble *et al.,* 2010; Víkingsson, 1995). Muscle lipid content is highly variable, ranging between 0.1 and 74.3% in our review, and is dependent on age, species, season and diet (Figure 3; Beck *et al.,* 1993; Mourot *et al.,* 2001; Trumble *et al.,* 2010; Shingfield *et al.,* 2013), further indicating the potential importance of skeletal muscle as an energy reserve tissue.

The body compartment most associated with lipid storage is adipose tissue, which takes the form of subcutaneous blubber, the specialized hypodermis in marine mammals. In addition to the lipids found in blubber, this body compartment contains water that varies inversely with lipid content (Dunkin *et al.,* 2005), variable amounts of protein (1.45–35%; Watanabe and Suzuki, 1950; Lockyer *et al.,* 1984; Lockyer, 1991; Gales *et al.,* 1994; Koep *et al.,* 2007; Víkingsson *et al.,* 2013; Anezaki *et al.,* 2016) and minimal amounts of carbohydrate (1–6%; Lockyer, 1991). Blubber also serves for streamlining, locomotion and thermoregulation, and these uses affect its lipid content (Worthy and Lavigne, 1987; Pabst *et al.,* 1999; Trumble *et al.,* 2010). Deep-diving sperm and beaked whales also incorporate wax esters into their blubber (Koopman, 2007; Bagge *et al.,* 2012). Although the role of wax esters in marine mammals is not fully known, it is posited that they do not aid in energy storage as they are more difficult to metabolize in vertebrates (Koopman, 2007; Koopman, 2018).

Additional energy reserves may exist in the viscera and bone (Lockyer *et al.,* 1985; Víkingsson, 1995). Lipids in the viscera range widely between 0.33 and 96% (from values reported for kidney, liver and heart), but it is not known what proportion of this lipid content is used for storage as some lipids may be structural (e.g. phospholipids). In the literature, visceral fat stores have been documented in whales that have already amassed large blubber stores (Lockyer, 1986). Anecdotal observations indicate depleted visceral fat stores in other cetaceans and pinnipeds that are nutritionally compromised (pers. comm. R. Dunkin and D. Rosen). The lipid content of bones ranges between 21.1 and 25.9%, however it is unknown what proportion of this is used to store excess lipids (Lockyer *et al.,* 1985; Víkingsson, 1995).

### Energy allocation to growth and energetic costs

The cost of depositing structural or reserve tissues, hereby known as the cost of growth, is believed to be small relative to total energy expenditure for most mammals (e.g. Roberts and Young, 1988; Víkingsson, 1995; Dalton *et al.,* 2015; Hin *et al.,* 2019; Christiansen *et al.,* 2022a). To measure the cost of growth, the excess energy above maintenance required to deposit tissues must be uncoupled from other metabolic processes such as locomotion, digestion and thermoregulation. Due to the difficulties of isolating growth costs, available estimates have been obtained using a variety of methods and assumptions. Some methods use only the energy content of the deposited tissues to estimate the total cost of growth (i.e. 1 kJ of energy is required to deposit 1 kJ of tissue), which does not account for the inefficiencies (secondary costs related to the chemical energy required to synthesize tissues) associated with tissue synthesis and deposition. Studies from other mammals that incorporate these inefficiencies, including from rats, pigs and cattle, estimate the energetic cost of tissue deposition to range from approximately 1.17 to 1.37 kJ/kJ for fat and 1.92 to 2.38 kJ/kJ for protein (Roberts and Young, 1988). Because of the difference in deposition costs between fat and protein, as well as the higher energy density of lipid, tissues with a higher lipid content (e.g. blubber) require more ingested energy and higher metabolic costs to deposit.

Alternately, the cost of tissue deposition, incorporating energetic inefficiencies, can be estimated by increasing energy intake above maintenance energy intake and measuring concurrent changes in mass (Blaxter, 1968). This technique yields a total cost of growth ranging from 12.2 kJ/g to a maximum of 63 kJ/g in small mammals, humans and some birds depending on the composition of growth and how growth inefficiencies are calculated (Millward *et al.,* 1976; Rattray and Joyce, 1976; Pullar and Webster, 1977; Roberts and Young, 1988). However, these methods have yet to be applied to marine mammals.

In most cases, the cost of growth in marine mammals has been estimated using the energy density of protein and lipid and the mass of deposited tissues, without accounting for inefficiencies. For example, this method was used to estimate the cost of growth in southern right whales (*Eubalaena australis*) resulting in costs of 27.163 kJ/g for blubber and 9.732 kJ/g for skeletal muscle (Christiansen *et al.,* 2022a). When using this method it is important to remember that skeletal muscle and blubber contain both protein and lipid (i.e. skeletal muscle is not solely protein). Though this can be avoided when using total body protein and lipid content. Additionally, the reported energy density values range from 19.66–26.6 kJ/g for protein and 37.66–39.75 kJ/g for lipid (Kleiber, 1947; Brody, 1968). The energy density of protein differs depending on its use for tissue synthesis or catabolism and at what point in the digestion to deposition chain it is accounted for. The energy density of protein is 26.6 kJ/g, but after oxidation to CO_2_, water and ammonia this is reduced to 23.43 kJ/g (Kleiber, 1975). Once protein is metabolized, creating CO_2_, water and urea, the energy density is reduced to generate 19.66 kJ/g, which pre-accounts for the chemical energy lost in urine as urea (Kleiber, 1961). As such, it is important to understand what the selected energy density value represents (i.e. pre- or post-metabolized protein).

### Empirical estimate of growth costs

In marine mammals, there have been few attempts to empirically estimate the total cost of growth using metabolic rate or energy intake. An approximation of the cost of protein deposition in northern fur seals has been proposed as 7% of daily energy expenditure for postweaning females (Dalton *et al.,* 2015), while Atlantic fin whales must consume 2–3% of body weight in prey to both meet metabolic demands and add additional energy reserves (Víkingsson, 1995). When examining resting metabolic rate in positive and negative growth phases, we found a significant increase in metabolic rate of northern fur seals during positive growth phases (Wilcoxon signed rank test, *P* = 2.4 × 10^−3^; Figure 3). There was no significant difference in resting metabolic rate in positive and negative growth phases in bottlenose dolphins likely because these individuals were adults and therefore only depositing small lipid stores in contrast to the greater lipid and protein deposition of juveniles in the primary growth phase. Further, the minimal increase in resting metabolic rate in positive growth phases for bottlenose dolphins may be a result of metabolic compensation to decrease the overall energetic strain of depositing new tissues.

**Figure 4:**
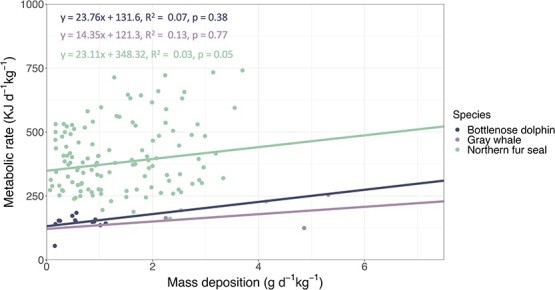
Linear mixed effect model of mass deposition normalized by body size (g d^−1^ kg^−1^) and resting mass-specific metabolic rate (kJ d^−1^ kg^−1^) for juvenile bottlenose dolphins (*n* = 2), northern fur seals (*n* = 6) and a single gray whale with random effects of individuals for northern fur seals. Data contributed by the University of California Santa Cruz Long Marine Laboratory and the University of British Columbia Marine Mammal Research Unit, and SeaWorld.

When examining the relationship between mass deposition and metabolic rate, the best model included mass-specific metabolic rate and mass deposition normalized by body size for all species, with a significant random effect of individual only for northern fur seals (AIC = 1559.31, 129.51 and 38.09 for northern fur seals, bottlenose dolphins and the gray whale, respectively). Mass-specific metabolic rate increased with mass deposition across all species, resulting in an estimated cost of growth of 23.11, 23.76 and 14.35 kJ/g, for northern fur seals, bottlenose dolphins and the gray whale, respectively (Figure 4). The estimated cost of growth derived in this study is within the range of reported for other mammals (e.g. Millward *et al.,* 1976; Rattray and Joyce, 1976; Pullar and Webster, 1977; Roberts and Young, 1988; Christiansen *et al.,* 2022a). Interestingly, juvenile northern fur seals had a similar cost of growth to adult bottlenose dolphins which may indicate that the composition of tissues deposited are similar, despite the difference in species and age class. The gray whale calf had a much lower estimated cost of growth. This may be an artifact of the sampling method, which used gross energy intake from prey as a proxy for metabolic rate. Alternately, this may indicate that very large animals, such as the gray whale, have proportionally lower costs of growth per their size. However, further investigation into this topic is warranted.

### Factors influencing total body size

#### Maternal investment

Maternal investment in offspring is vital to growth and development as young animals are fully or partially dependent on milk until they reach weaning age. Maternal mass is highly correlated with offspring mass at both birth and weaning across mammals (Bowen *et al.,* 2015; Holser *et al.,* 2021; Allen *et al.,* 2022; Christiansen *et al.,* 2022a; Costa and Maresh, 2022). Females in better condition yield larger, presumably healthier offspring (e.g. Kovacs and Lavigne, 1986; Taillon *et al.,* 2012; Christiansen *et al.,* 2018; Dias *et al.,* 2018; Holser *et al.,* 2021; Stewart *et al.,* 2022). This pattern holds true for both pinnipeds and cetaceans (e.g. Kovacs and Lavigne, 1986; McDonald *et al.,* 2008; Christiansen *et al.,* 2014; Christiansen *et al.,* 2018) and is likely the result of the relationship between female condition and fetal growth, as well as the relationship between milk quality and quantity and early calf development (e.g. Festa-Bianchet *et al.,* 1998; Georges and Guinet, 2000; West *et al.,* 2007; Costa, 2008; Riet-Sapriza *et al.,* 2012). So important is this relationship between maternal condition and offspring growth that populations may decrease if maternal body condition is chronically poor amongst females. For example, calf growth rates of North Atlantic right whales (*Eubalaena glacialis*) have declined in conjunction with population-wide decreases in maternal body condition (Christiansen *et al.,* 2020; Stewart *et al.,* 2021). Additionally, plasticity in maternal care allows females to allocate resources either to their current reproductive effort or future reproductive success, affecting how young, nutritionally dependent animals grow (Boness *et al.,* 1991; Festa-Bianchet *et al.,* 1998; Costa, 2008; McMahon *et al.,* 2017).

#### Population dynamics

In vertebrates, density-dependence, increased competition or predation and mortality risks can affect prenatal stress on the mother which can alter pre- and postnatal growth in offspring (Coslovsky and Richner, 2011; Dantzer *et al.,* 2013; Berghänel *et al.,* 2017; Holser *et al.,* 2021). Some populations experience increases in offspring growth rates as populations near carrying capacity, because larger individuals are superior competitors for resources (Coslovsky and Richner, 2011; Dantzer *et al.,* 2013). However, individual growth rates have also been shown to decline in response to increasing density-dependence (Kato, 1987; Fowler, 1990; Harding *et al.,* 2018), likely due to a decrease in per-capita environmental resources as populations reach carrying capacity.

#### Environment

Seasonal and annual changes in prey availability and temperature affect the overall energy budget of animals, resulting in unique species-specific changes in energy allocation to growth. For example, during El Niño events, when decreased prey availability in some regions yields reduced energy intake, Galapagos fur seal (*Arctocephalus galapagoensis*) pups were lighter in weight and California sea lion pups were smaller than their predicted size-at-age (Trillmich and Limberger, 1985; Boness *et al.,* 1991). In other species decreased energy intake may not influence growth. For example, Steller sea lions (*Eumetopias jubatus*) maintained structural growth even when fed low energy density diets (Rosen *et al.,* 2006). However, it is important to note that these Steller sea lions experienced short-term reductions in energy intake in human care. Longer-term periods of decreased energy intake could have more severe negative influences on growth because there is limited evidence that pinnipeds can demonstrate compensatory growth to make up for earlier nutritional challenges (du Dot *et al.,* 2008).

The relationship between the environment and growth have been described using ecogeographic rules; Bergmann’s rule and McNab’s rule. Bergmann’s rule and McNab’s rule describe a latitudinal increase in overall body size within and amongst species (Bergmann, 1847; Mayr, 1956; McNab, 1971; McNab, 2010). Bergmann’s rule posits that larger body size near the poles is driven by greater thermoregulatory needs. Larger organisms have a lower surface area to volume ratio and, therefore, less heat is lost per unit of heat that is produced (McNab, 1971). However, McNab’s rule posits that this latitudinal trend may be driven by differences in resource availability. Lower latitudes have lower prey densities, favoring smaller body sizes (McNab, 2010). Latitudinal gradients in marine mammal body size attributed to both Bergmann’s and McNab’s rules have been identified at broad taxonomic levels and between closely related species (Galatius and Gol’din, 2011; Oosthuizen *et al.,* 2016; Torres-Romero *et al.,* 2016; Best *et al.,* 2017; Ferguson *et al.,* 2018; van Aswegen *et al.,* 2019; Adamczak *et al.,* 2020). For example, closely related pilot whale (Globicephala spp.) species differ slightly in body size and surface area to volume ratio, with the larger, more northern species being better equipped for the colder waters within its range, supporting Bergmann’s rule (Adamczak *et al.,* 2020). In support of McNab’s rule, harbor porpoise populations along the California coast are larger than other populations, which may be driven by the nutrient-rich upwelling in this region (Galatius and Gol’din, 2011). Additionally, latitudinal gradients in sperm whale body size have been linked to changes in prey size along this gradient (Best *et al.,* 2017). Similar trends are seen when comparing the recently diverged California sea lion and Galápagos sea lion (*Zalophus wollebaeki*). California sea lions are larger in overall size and inhabit more productive northern waters of the Pacific Ocean, which may be driven by latitudinal gradients in food availability rather than genetics (Schramm *et al.,* 2009; Villegas-Amtmann *et al.,* 2011). Although an empirical test of the contribution of genetics or environment to size clines in marine mammals has not been carried out, conclusions from other vertebrates show contrasting results, highlighting the complexity of this question (Teplitsky *et al.,* 2008; Husby *et al.,* 2011; Ballinger and Nachman, 2022).

### Factors influencing energy reserves

#### Life history events

Reproduction is a period of increased energy expenditure for marine mammals, resulting in changes to body energy reserves (Costa *et al.,* 1989; Arnould and Duck, 1997; Crocker *et al.,* 2012; Bejarano *et al.,* 2017; Christiansen *et al.,* 2022b). However, the nature of investment into deposition and utilization of reserves differs with breeding strategy (Costa and Maresh, 2022). Capital breeding females store proportionally more energy than income breeders of similar size (Irvine *et al.,* 2017) and increase lipid reserves prior to pregnancy (Lockyer, 1986; Miller *et al.,* 2011; Lemos *et al.,* 2020). These lipid reserves are then quickly depleted during lactation as the female typically fasts during this period (Miller *et al.,* 2011; Richard *et al.,* 2014; Pettis *et al.,* 2017; Lemos *et al.,* 2020; Aoki *et al.,* 2021). Protein catabolism during lactation also plays an important role in fluctuations of overall body mass in phocids, particularly when lipid reserves are depleted (e.g. Crocker *et al.,* 1998). In contrast, female income breeders typically exhibit a much slower and steadier decrease, or in some cases no decrease, in mass and condition throughout lactation (Costa *et al.,* 1989). This is facilitated by simultaneous feeding over much longer lactation durations (i.e. months to years) when compared to capital breeders (i.e. days to months; Perrin and Reilly, 1984; Read and Hohn, 1995; McDonald and Crocker, 2006; Karniski *et al.,* 2018; Costa and Maresh, 2022). Many marine mammals exist on a continuum from capital to income breeders, and others exhibit a mix of these strategies, necessitating different lipid sequestration during reproduction (Noren *et al.,* 2014).

Males also incur reproductive costs that influence body reserves, despite the differing reproductive strategies between the sexes (Arnould and Duck, 1997;Coltman *et al.,* 1998; Crocker *et al.,* 2012). This has predominantly been studied in pinnipeds due to the difficulties associated with studying breeding and reproductive costs in fully aquatic species. In pinnipeds, male reproductive costs are typically incurred via territory defense during which many species fast to maintain a territory or harem (e.g. Anderson and Fedak, 1985; Boyd and Duck, 1991; Bartsh *et al.,* 1992; Arnould and Duck, 1997). However, some species that reproduce in the water, such as the harbor seal, may opportunistically feed throughout the breeding season, offsetting large declines in body mass (Coltman *et al.,* 1998). Across most pinniped species, larger males lose proportionally more body mass throughout the breeding season in exchange for greater mating success and more breeding opportunities (Anderson and Fedak, 1985; Deutsch *et al.,* 1990; Coltman *et al.,* 1998; Crocker *et al.,* 2012). While defending territories males lose primarily lipid and attempt to conserve protein (Coltman *et al.,* 1998; Crocker *et al.,* 2012).

Molting is an essential life-history event that can also result in a negative energy balance and changes in body mass. Although some cetaceans do molt their skin (St. Aubin *et al.,* 1990; Pitman *et al.,* 2020), the literature on reserve utilization while molting is dominated by pinnipeds that shed fur. Declines in overall body mass are observed in molting pinnipeds attributable to decreases in both lipid and protein reserves (Worthy *et al.,* 1992; Boyd *et al.,* 1993; Noren *et al.,* 2003; Noren and Mangel, 2004; Field *et al.,* 2005; Williams *et al.,* 2007). Although there may be increased metabolic expenditure related to pelage growth and thermoregulation during the molt (Boily, 1995; Boily, 1996; Pitman *et al.,* 2020; Pearson *et al.,* 2022), the primary cause for decreased reserves in phocids is a reduction in feeding as these animals often haul-out to molt (Thometz *et al.,* 2021).

Similarly, marine mammal migration causes increased metabolic demand from traveling long distances coupled with decreased foraging opportunities and feeding events (Alexander, 1998; Alerstam *et al.,* 2003), likely resulting in declines in overall body size through mobilization of reserves. Migration can cover distances up to 8000 km, such as the humpback whale (*Megaptera novaeangliae*) migration from the cold feeding grounds to warm breeding grounds, during which the animals will not forage or will forage minimally (Corkeron *et al.,* 2019). Amongst migrating cetaceans, typically the larger species with greater energy reserves travel the greatest differences (Boyd, 2004). Even within a species, larger individuals within a species can travel greater distances with fewer consequences on their overall body size and energy reserves (Boyd, 2004).

#### Prey availability and composition

Prey availability and quality can also influence an animal’s energy balance, requiring reliance on energy reserves in resource-poor environments. In mysticetes, lipid reserves typically fluctuate annually in response to prey availability, often decreasing when prey quality and quantity is low (Haug *et al.,* 2002; Konishi *et al.,* 2008; Miller *et al.,* 2011; Williams *et al.,* 2013; Braithwaite *et al.,* 2015; Lemos *et al.,* 2020). Similarly, phocids typically have larger lipid reserves, often expressed as improved body condition, when inhabiting more productive environments than conspecifics in other habitats (Bailleul *et al.,* 2007; Arce *et al.,* 2022). Although seasonal fluctuations in energy reserves are observed in otariids and odontocetes, there is little documentation of how these seasonal fluctuations relate to prey availability. Seasonal fluctuations in energy reserves with prey availability may be more apparent in capital breeders than income breeders as they have a proclivity to amass proportionally greater energy reserves when prey are abundant (Stewart and Lavigne, 1984; Stephens *et al.,* 2014; Irvine *et al.,* 2017). Relating environmental state, resource availability and animal performance presents a challenge in ecology, but new tools, software and in situ communication systems can help inform these transfer functions and are the focus of much attention.

Diet and prey composition also influence lipid reserves in both blubber and muscle; however, the observed trends can be complex. In mysticetes, more lipid-rich prey often yields higher lipid content in blubber and muscle, although the majority of this work has been completed in field experiments and, as such, it is unknown if changes to diets were isocaloric (Næss *et al.,* 1998; Víkingsson *et al.,* 2013). However, in phocids and otariids, the influence of diet on lipid reserves is unclear. High-lipid diets do not consistently increase lipid reserves in phocids, while low-lipid diets—particularly during nutritional challenges—result in a loss of lipid reserves (Kirsch *et al.,* 2000; Rosen and Trites, 2000; Trumble *et al.,* 2003); however, only one of these studies held calorie content of intake constant, potentially obscuring results. As such, the role of diet in lipid deposition is somewhat complex and is driven by several intrinsic and extrinsic factors.

#### Environment

Variation in environmental temperature also affects the accumulation and utilization of lipid reserves. In colder waters, dolphins increase blubber thickness (Noren and Wells, 2009) and may store more lipids (e.g. Montie *et al.,* 2008); however, this is likely driven by thermoregulatory needs rather than storage needs. Additionally, in regions with large temperature fluctuations, animals appear to store more lipid in preparation for or in response to increased thermoregulatory demands (e.g. Lockyer *et al.,* 2003; Adamczak *et al.,* 2021). Indeed, in controlled studies in human care, body mass and blubber thickness of adult female walruses tracked air temperature, where during warmer periods walruses decreased their food consumption and body mass while blubber thickness decreased (Noren *et al.,* 2015). Furthermore, body mass and blubber thickness of pilot whales tracked water temperature where a dramatic drop in water temperature resulted in increased food consumption and increased body mass and blubber thicknesses (Noren *et al.,* 2021).

#### Body condition thresholds

Linking energy reserve levels to fitness is a key component of many bioenergetic models. In many models, a minimum body condition threshold is often set to 5% body fat (e.g. Malavear, 2002; Beltran *et al.,* 2017; Pirotta *et al.,* 2018b; Gallagher *et al.,* 2021. This value is derived from pigs and is assumed to be the lowest possible fat level that allows for basic metabolic functioning (Whittemore, 1998). Although the use of this 5% body fat minimum may provide a conservative estimate of when individuals die due to lack of energy stores, it is probable that mortality occurs before this threshold is reached and that declining body condition could progressively increase the probability of mortality or decrease the probability of calving. Anecdotal evidence from stranded and sick animals could be used to define a more realistic minimum body condition threshold for marine mammals. There is sufficient data on stranded marine mammals to compute this threshold; however, these minimum values are often not reported in the literature. A potential body fat minimum derived from a sick northern elephant seal (*Mirounga angustirostris*) that died shortly after morphometric measurement were taken is 18.3%, which is compared to an average of 30.4% in healthy animals (Holser *et al.,* 2023). In addition to minimum body storage levels for survival, we can assume that there are minimum body storage levels for successful pregnancy, lactation and other reproductive events (Laws, 1956). However, it is difficult to empirically obtain estimates of those thresholds as it would require monitoring of the storage levels amongst individuals that successfully reproduce and those that do not, which requires long-term monitoring datasets and data collection of behavior, reproductive histories and morphometrics.

## Data Gaps

Measurements of structural mass and energy reserves provide relevant individual and population health proxies and are essential input parameters for bioenergetic models. Despite the importance of these data, there are many species and species groups without adequate growth curves (e.g. river dolphins and beaked whales) and impacts of resource restriction on projected growth curves are difficult to obtain. Evaluating how growth curves from data-rich species can be applied or adjusted to fit the growth of data-poor species can be a valuable tool to fill in gaps for data-poor species. Further, drones and remote three-dimensional imagery provide exciting new opportunities to acquire data on these otherwise hard-to-study species. To address alterations in growth in response to climate and resource-related changes, long-term datasets and long studied populations can begin to elucidate these patterns and may help us target populations that are at risk of decline (e.g. Christiansen *et al.,* 2020). As such, when assessing growth curves in species and populations, it is important to also consider the current population size and population trajectory (i.e. growing, stable, declining) at the time of sampling when possible and if this is representative of current conditions. This will provide baseline information from which we can estimate population health based on growth rates of individuals within a population.

Understanding the preferential storage and mobilization of reserves to different body compartments will allow better estimates of total body lipid and protein reserves. The contribution of protein reserves to critical life-history events, such as molting and lactation, is important to document as the current marker for individual health typically focuses on the size of lipid stores. This is particularly relevant when relying on external morphology (i.e. how wide an animal is) or blubber biopsies to provide a proxy of lipid reserves, as these might not always be representative of the overall energy reserves of the animals (Kershaw *et al.,* 2019; Christiansen *et al.,* 2020). While data on protein catabolism exist for a few pinnipeds, with much of the otariid literature focusing on protein catabolism during post-weaning fasts (Nordøy *et al.,* 1990; Oftedal 1997; Rea *et al.,* 2000), data on cetaceans have only recently been collected with bottlenose dolphins as the primary study species (e.g. Suzuki *et al.,* 2018; Houser *et al.,* 2021; Derous *et al.,* 2022). There are sufficient data to indicate that the interaction between protein and fat content of the diet is complex and deserves further investigation, particularly in the context of bioenergetic models where energy stores are predominantly assumed to be derived from lipids. The advent of metabolic markers and omics technologies provides an opportunity to improve our understanding of how stored energy is mobilized and which stores are preferred during different life-history events (e.g. Derous *et al.,* 2022).

Improving estimates of the cost of growth in marine mammals will improve modeled growth costs in bioenergetic models and provide context for how growth is altered when resources are limited. Until we can empirically measure the cost of growth, we can improve our understanding of these costs with direct energy density measurements for protein and lipid from marine mammals. Surprisingly, little data on this topic have been collected. The methodologies are established and can be used on opportunistic stranded and bycaught animals or biopsy samples. Although the discrepancies between energy density values appear to be relatively small, there can be considerable consequences when estimating growth costs for larger species that must deposit proportionally more protein and lipid.

## Funding

Funded under award from Office of Naval Research: N000142012392. DPC and SA were funded under the E&P Sound and Marine Life Joint Industry Programme of the International Association of Oil and Gas Producers (IOGP; grant 00-07-23). CRM is supported by the Australian Integrated Marine Observing System (IMOS), IMOS s enabled by the National Collaborative Research Infrastructure Strategy.

## Data Availability

The data compiled for this review on tissue composition and available growth curves are published in the supplementary material of this manuscript and are listed as Supplementary Table A and Supplementary Table B, respectively.

## Author Contributions

S.K.A., E.A.M. and D.P.C. conceived the manuscript and S.K.A. led the writing of the manuscript. R.D., D.R. and J.S. contributed data for analyses. F.C., C.R.M., S.N. and E.P. contributed critically to the drafts and revisions of the manuscript. All authors read and approved the final manuscript.

## Supplementary material

Supplementary material is available at *Conservation Physiology* online.

## Supplementary Material

Web_Material_coad035
